# Ultra Q-bodies: quench-based antibody probes that utilize dye-dye interactions with enhanced antigen-dependent fluorescence

**DOI:** 10.1038/srep04640

**Published:** 2014-04-11

**Authors:** Ryoji Abe, Hee-Jin Jeong, Dai Arakawa, Jinhua Dong, Hiroyuki Ohashi, Rena Kaigome, Fujio Saiki, Kyosuke Yamane, Hiroaki Takagi, Hiroshi Ueda

**Affiliations:** 1 Ushio Inc., 6409 Moto-Ishikawa-cho, Aoba-ku, Yokohama, Kanagawa 225-0004, Japan; 2 Department of Chemistry and Biotechnology, School of Engineering, The University of Tokyo, 7-3-1 Hongo, Bunkyo-ku, Tokyo 113-8656, Japan; 3 Chemical Resources Laboratory, Tokyo Institute of Technology, 4259-R1-18, Nagatsuta-cho, Midori-ku, Yokoyama, Kanagawa 226-8503, Japan

## Abstract

Recently, we described a novel reagentless fluorescent biosensor strategy named Quenchbody, which functions via the antigen-dependent removal of the quenching effect on a fluorophore that is attached to a single-chain antibody variable region. To explore the practical utility of Quenchbodies, we prepared antibody Fab fragments that were fluorolabeled at either one or two of the N-terminal regions, using a cell-free translation-mediated position-specific protein labeling system. Unexpectedly, the Fab fragment labeled at the heavy chain N-terminal region demonstrated a deeper quenching and antigen-dependent release compared to that observed using scFv. Moreover, when the Fab was fluorolabeled at the two N-termini with either the same dye or with two different dyes, an improved response due to enhanced quenching via dye-dye interactions was observed. On the basis of this approach, several targets, including peptides, proteins, and haptens, as well as narcotics, were quantified with a higher response up to 50-fold. In addition, differentiation of osteosarcoma to osteoblasts was successfully imaged using a similarly fluorolabeled recombinant Fab protein prepared from *E. coli*. Due to its versatility, this “Ultra-Quenchbody” is expected to exhibit a range of applications from *in vitro* diagnostics to the live imaging of various targets *in situ*.

Protein-based fluorescent probes are widely used for the specific detection of a trace amount of substances *in vivo* and *in vitro*. However, to date, most of these developed probes (biosensors) rely on the function of a target-specific natural receptor, and each construct must be designed and empirically and individually tested for its performance, which is a laborious and time-consuming process.

As a possible solution for this issue, we recently reported a general strategy for the detection of a broad range of biomolecules using a fluorolabeled antibody fragment called “Quenchbody (Q-body)”^1^. The fluorescence of Q-body (a position-specific fluorolabeled single-chain variable region, scFv) was quenched in its antigen-free state due to the interaction (photoinduced electron transfer, PET) of the intramolecular tryptophan (Trp) residues to the attached dye, i.e., TAMRA. However, once Q-body binds to its target antigen, the quenching interaction is released by an antigen-induced conformational change, resulting in increased fluorescence of the exposed dye from the antibody. This increase in fluorescence is rapid and reflects the target antigen concentration. Unlike other fluorescence-based probes, including reagentless biosensors^2^,^3^,^4^, Q-bodies may be generated for a range of targets, from small molecules such as narcotics to larger proteins, such as serum albumins, on the basis of this assay principle.

However, despite these merits, some Q-bodies exhibit a limited fluorescence response and/or sensitivity, which is most likely due to insufficient Trp-mediated quenching of the attached dye in the absence of antigen. Moreover, the stability and affinity of scFv, compared with its parental full-size antibody or fragment of antigen binding (Fab), is of controversial concern^5^,^6^. To resolve this problem, we propose a new strategy to generate Fab-based Q-bodies, which incorporates the use of multiple dyes. Our results clearly demonstrate its superior performance compared to scFv-based Q-bodies in both detection sensitivity and response. Furthermore, as an application to bioimaging, identification of osteoblasts was successfully performed using Fab-based Q-body, which was successfully prepared from a recombinant protein expressed in *E. coli*.

## Results

### Generation of Fab-type Q-bodies

Because Fab fragments are considered more thermostable compared to the single chain variable region (scFv), which had been successfully converted to a Q-body, we tried to synthesize Q-bodies based on Fab fragments. As a model antibody, we selected anti-human osteocalcin (bone gla protein, BGP) KTM219 as the recognition unit for the bone-related disease marker^7^. TAMRA was the dye used to conjugate with the Fab as previously described.

**Figure 1:**
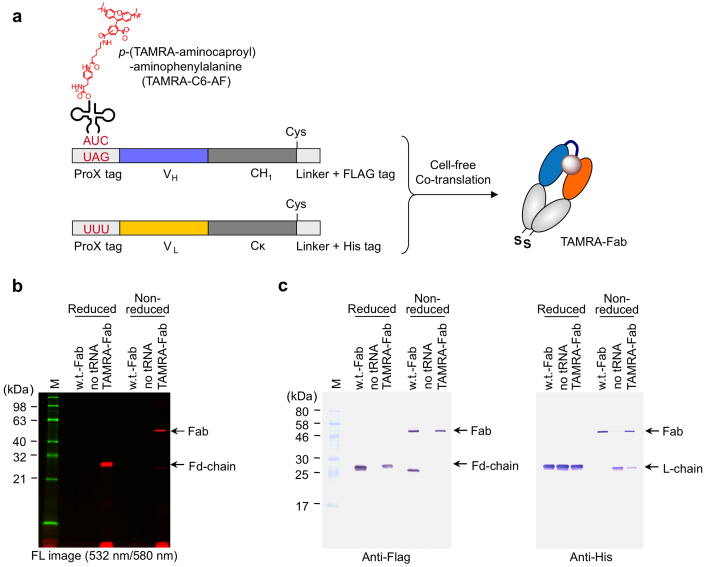
Synthesis of the Fab-containing TAMRA at the N-terminal region of the H chain. (a) Scheme of the incorporation of TAMRA-C6-AF into the H chain (Fd, V_H_-C_H_1) of Fab in response to a UAG codon in a cell-free translation system. (b) SDS-PAGE fluorescence image of the expressed anti-BGP Fab containing the TAMRA-C6-AF in reducing and non-reducing conditions. TAMRA fluorescence was detected with an excitation at 532 nm and emission at 580 nm. The fluorescent marker shown in green was detected with an excitation at 488 nm and emission at 520 nm. (c) Western blotting analysis of the same gel using anti-Flag and anti-His antibodies.

To synthesize Fab-type Q-bodies, we employed two plasmids encoding either V_H_-C_H_1 (Fd chain) or C_L_-Cκ (L chain) cDNAs, and incorporated TAMRA into the N-terminal ProX tag region of the Fd during cell-free co-transcription-translation. In addition, Flag- and His-tags were fused at the C-terminus of the Fd and L chains, respectively, to enable their detection and tandem purification (Fig. 1a). After running the translated products on a 15% SDS-PAGE gel, the synthesized anti-BGP Fab protein was detected using gel fluorescence imaging (Fig. 1b). A clear fluorescent band reflecting TAMRA fluorescence was observed at the expected molecular weight (26 kDa) of the Fd chain in the reduced sample, and the heterodimeric Fab (Fd/L) was observed at 53 kDa in the non-reduced, denatured sample. This result indicated that a heterodimeric Fab fragment incorporated with TAMRA at the N-terminal region of Fd was successfully generated. The Fab was also detected using Western blotting analyses with anti-Flag and anti-His antibodies. Heterodimeric Fab was observed in the presence of TAMRA-C6-AF-tRNA, although a negligible amount of protein was observed in the absence of suppressor tRNA (Fig. 1c). Taken together, these results indicated that TAMRA-C6-AF-tRNA specifically decoded the amber codon. Furthermore, the anti-BGP Fab was site-specifically and quantitatively labeled with TAMRA.

### Antigen-dependent fluorescence of TAMRA-labeled anti-BGP Fab

**Figure 2:**
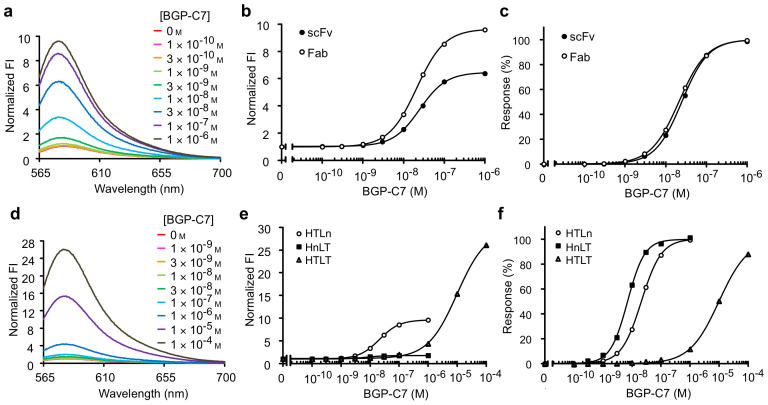
Antigen-dependent fluorescence enhancement of TAMRA-labeled anti-BGP Fab. (a) Fluorescence spectra of TAMRA-scFv with an excitation at 550 nm in the absence and presence of BGP-C7 peptide. (b) Standard curve of the fluorescence intensity at 580 nm. The intensities are the relative values in the absence of BGP-C7 peptide. (c) Normalized standard curves. (d) Fluorescence spectra of double TAMRA-Fab with an excitation at 550 nm in the presence of BGP-C7 peptide as shown. (e) Standard curve of the fluorescence intensity at 580 nm. The intensities are the relative values to that in the absence of BGP-C7 peptide. (f) Normalized dose-response curves.

The labeled Fab was purified using tandem affinity purification with anti-Flag and Ni-NTA resins. Its fluorescence spectra were measured in the presence of various concentrations of antigen BGP-C7 peptide (NH_2_-RRFYGPV-COOH) (Fig. 2, a–c), resulting in a remarkable dose-dependent increase (9.6-fold) in the intensity of the fluorescence spectra upon the addition of BGP-C7. This increase exceeded that of the scFv Q-body (5.6-fold). To investigate the reason of increased response, the effect of denaturant 7 M guanidium hydrochloride (GdnHCl) added with 100 mM DTT was investigated. Indeed, the labeled Fab showed higher fluorescence enhancement (9.5-fold) than the scFv (5.5-fold), indicating deeper quenching of Fab Q-body in the absence of antigen. A standard curve of the fluorescence intensity at 580 nm suggested that the fluorescence increased in the BGP-C7 peptide concentration occurred in a dose-dependent manner (Fig. 2b). Using curve fitting, the EC_50_ value of 2.1 × 10^−8^ M was obtained, which was lower compared to scFv (2.5 × 10^−8^ M). Both the deeper quenching and lower EC_50_ most likely reflected the enhanced stability of the Fab fragment, while maintaining similarly efficient antigen-dependent de-quenching property.

### Fluorescence-based thermal shift assay

In general, the Fab fragment retains a higher thermostability compared to the scFv^6^. To compare the thermostability properties of the scFv- and Fab-type Q-bodies, a fluorescence-based thermal shift assay was performed using a real-time thermal cycler^8^. Instead of using an external environmentally sensitive dye, we took advantage of the temperature-dependent denaturation and de-quenching (fluorescence recovery) properties of Q-bodies, as previously demonstrated in its urea-induced denaturation. As a result, melting temperature (*T*_m_) values of 73°C and 61°C were obtained for the Fab- and scFv-type Q-bodies, respectively (Supplementary Fig. S1). The observed higher thermostability of Fab-type Q-body was most likely due to the tighter association of C_H1_ and C_L_, which are connected by a C-terminal disulfide bond.

### TAMRA double-labeled Q-body demonstrates a reduced sensitivity due to the H-dimer

Unlike the scFv fragment, the Fab fragment has two N-termini, each at the Fd and L chains, respectively. To investigate whether a higher fluorescent response can be attained with dye-dye quenching, an expression construct was generated in which an additional amber codon was incorporated into the ProX tag region of the L chain, to attach two TAMRA molecules per Fab. The obtained doubly TAMRA-labeled Q-body, shown as H-TAMRA/L-TAMRA (HTLT), demonstrated antigen-dependent fluorescence intensity increases with a maximum of 25-fold under the same conditions, suggesting deeper fluorescence quenching in the absence of antigen (Fig. 2, d–f). However, the detection sensitivity of this Q-body was very modest with an EC_50_ of 1.0 × 10^−4^ M, which is a value that well exceeded that of single TAMRA-labeled Q-bodies. Indeed, the sensitivities of the H-TAMRA/L-nonlabel (HTLn) and H-nonlabel/L-TAMRA (HnLT) were both higher with an EC_50_ of 2.1 × 10^−8^ M and 7.1 × 10^−9^ M, respectively. Thus, we postulated that the strong interaction, known as H-dimer formation^9^, between the two TAMRA dyes competes with the antigen binding and subsequent closure of the V_H_/V_L_ interface, thereby resulting in markedly reduced sensitivity. As fluorescent dyes that are potentially more resistant to H-dimer formation, we attempted to incorporate two other fluorescent dyes, rhodamine 110 (R110) and ATTO655. We found that the fluorescence spectra of double R110- and ATTO655-labeled Fab Q-bodies, namely, H-R110/L-R110 (HRLR) and H-ATTO655/L-ATTO655 (HALA), demonstrated enhanced BGP-C7 dose-dependent fluorescence increases (Supplementary Fig. S2). The maximal fluorescence responses of the HRLR and HALA Q-bodies were 2.4- and 11-fold, respectively, which were greater than that of single dye-labeled Fab Q-bodies and the corresponding scFv Q-bodies (Table 1). Responses of the HRLR and HALA Q-bodies were observed with an estimated EC_50_ of 7.6 × 10^−9^ M and 4.4 × 10^−8^ M, respectively, which were closer to that of single dye-labeled Fab proteins. The lower EC_50_ values than TAMRA double-labeled Fab probably reflected their lower dimerization/self-quenching behaviors.

Taken together, these results suggested that the doubly labeled Fab Q-bodies, in which the same dye was incorporated to both the H and L chains, resulted in deeper quenching and a greater antigen-dependent release, due to H-dimer formation and Trp-mediated PET. Because the performances of these Fab Q-bodies far exceeded those of conventional Q-bodies, these position-specific fluorolabeled Fabs were named “Ultra-Quenchbodies (UQ-bodies)”.

### Hetero double-labeled Fab Q-bodies demonstrate a higher response

Studies performed by Kajihara et al.^10^ have demonstrated the use of a position-specific labeling system, where two different dyes may be incorporated into two designated positions of a protein, using two types of specific codons, such as a four-base codon and an additional four-base or amber codon. As a result, a hetero double-labeled maltose binding protein can be successfully used to monitor changes in FRET that are dependent on a conformational change that occurs upon the binding of the substrate maltose.

**Figure 3:**
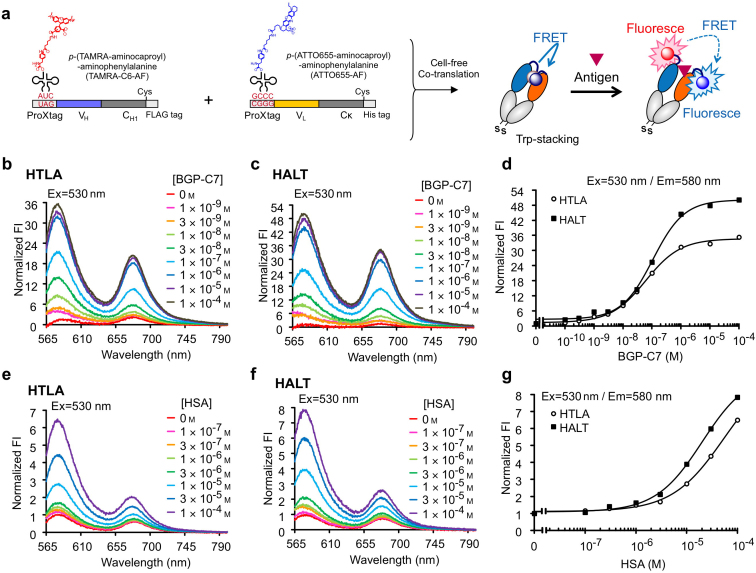
Antigen-dependent fluorescence enhancement of TAMRA/ATTO655-labeled Fab. (a) Scheme of the incorporation of TAMRA-C6-AF and ATTO655-C6-AF into the H and L chains of Fab in response to a UAG and 4-base codons in a cell-free translation system, respectively. (b) Fluorescence spectra of the Fab labeled at the N-terminal region of the H chain with TAMRA and the L chain with ATTO655 (HTLA), with excitation at 530 nm in the presence of BGP-C7 peptide as indicated. (c) The same with Fab labeled with ATTO655 at the H chain and with TAMRA at the L chain (HALT). (d) Standard curves of the fluorescence intensity at 580 nm. The intensities are the relative values to that in the absence of BGP-C7 peptide. (e–g) The results of the anti-SA UQ-bodies for detecting human serum albumin. The conditions were the same as in (b–d), except that HSA was used as an antigen.

As an extension of this approach, we incorporated two different dyes into one Fab molecule to further improve the FRET response between the dyes. To achieve this, we generated another pair of expression constructs in which the amber codon at the N-terminal ProX tag region was replaced with a CGGG codon, to incorporate two different fluorophores into a Fab during the translation reaction (Fig. 3a). We found that the fluorescence spectra of the FRET pair-labeled Q-bodies with the combination of TAMRA as a donor and ATTO655 as an acceptor produced high antigen responses (Fig. 3, b–d, Table 2). The maximum fluorescence responses of the HTLA and HALT Q-bodies at the donor emission wavelength (580 nm) were 50- and 35-fold, respectively, and maintained a high sensitivity. Since these values well exceed the Trp-mediated quenching of TAMRA measured by denaturation (9.5-fold), additional quenching by FRET is a plausible mechanism of the obtained high responses.

In addition, H-R110/L-TAMRA (HRLT) and H-TAMRA/L-R110 (HTLR) Q-bodies showed that the fluorescence derived from TAMRA increased in a dose-dependent manner upon the addition of BGP-C7 with an excitation of 480 nm. The maximum fluorescence responses of the HRLT and HTLR Q-bodies were 19- and 2.5-fold, respectively (Supplementary Fig. S3, Table 2). Probably, these values reflect the product of Trp- and FRET-mediated quenching of these dyes. After curve fitting, the EC_50_ values were 1.1 × 10^−7^ M and 9.7 × 10^−9^ M, respectively. In addition, fluorescence intensity ratios of 530 nm and 580 nm increased from 1.2 to 2.8, depending on the BGP-C7 concentration, with an EC_50_ value of 2.5 × 10^−8^ M.

### Application of the Ultra Q-body strategy using other Fab fragments

With the expectation of a broader application of the UQ-body strategy to other antibody/antigen pairs, we prepared UQ-bodies for other antigens. First, serum albumin (SA) was used as a model protein. To compare the performance of UQ-bodies to the conventional Q-body under the same conditions, UQ-bodies containing the (Gly_3_Ser)_2_ linker between the ProX tag and Fab were prepared as previously described^1^. As shown in Fig. 3, e–g, anti-SA UQ-bodies heterolabeled with TAMRA and ATTO655 showed a higher response of 6.5- to 7.8-fold while maintaining a similar sensitivity compared with previous scFv-type Q-bodies, which showed a modest response of up to 1.5-fold. The UQ-bodies labeled with R110 and TAMRA also demonstrated 3.5- to 8.3-fold maximum responses (Supplementary Fig. S3, d–f).

Next, the hemagglutinin (HA) protein of the influenza virus was selected as a target. We selected the human anti-influenza A HA antibody Fi6V3 due to its known structure as well as its high affinity to a broad range of influenza A viruses^11^. The heterolabeled UQ-bodies were constructed on the basis of synthetic V region genes, and we found 7-fold fluorescence increases upon the addition of H1N1 or H5N1 HA proteins (Supplementary Table S1, Supplementary Fig. S4).

### Application of the UQ-body strategy in drug detection

Previously, we have shown that the anti-morphine scFv Q-body could detect opiates in the ppb range by merely mixing the sample and measuring its fluorescence^1^. However, the maximum fluorescence increase was relatively modest (~1.5-fold). To obtain a more enhanced response, we generated UQ-bodies for morphine. In addition, the UQ-bodies for methamphetamine and cocaine were similarly prepared.

**Figure 4:**
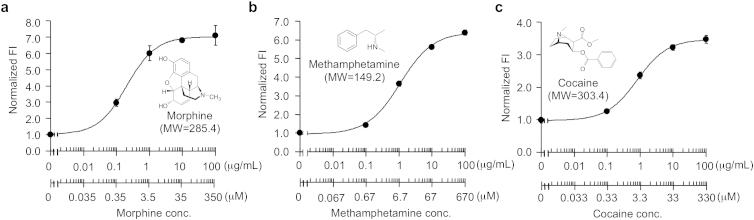
Titration curves of TAMRA-labeled anti-narcotics UQ-bodies. Anti-Morphine double-labeled Fab (a), anti-methamphetamine H chain-labeled Fab (b), and anti-cocaine double-labeled Fab (c) were used. Standard curves of fluorescence intensity at 580 nm with excitation at 530 nm are shown. Normalized fluorescence intensities show relative values with respect to that in the absence of antigen. An average with 1 SD of the eight samples is shown.

The fluorescence intensity for the purified proteins in the presence and absence of their respective antigen were evaluated using fluorescence spectral measurements. As shown in Fig. 4, all Fab Q-bodies showed an enhancement in antigen-dependent fluorescence. Among these enhancements, anti-morphine (HTLT), anti-methamphetamine (HTLn) and anti-cocaine (HTLT) showed the best antigen-dependent fluorescence responses (7.2-, 7.2- and 3.5-fold, respectively).

### Preparation of UQ-bodies using recombinant Fab fragments

**Figure 5:**
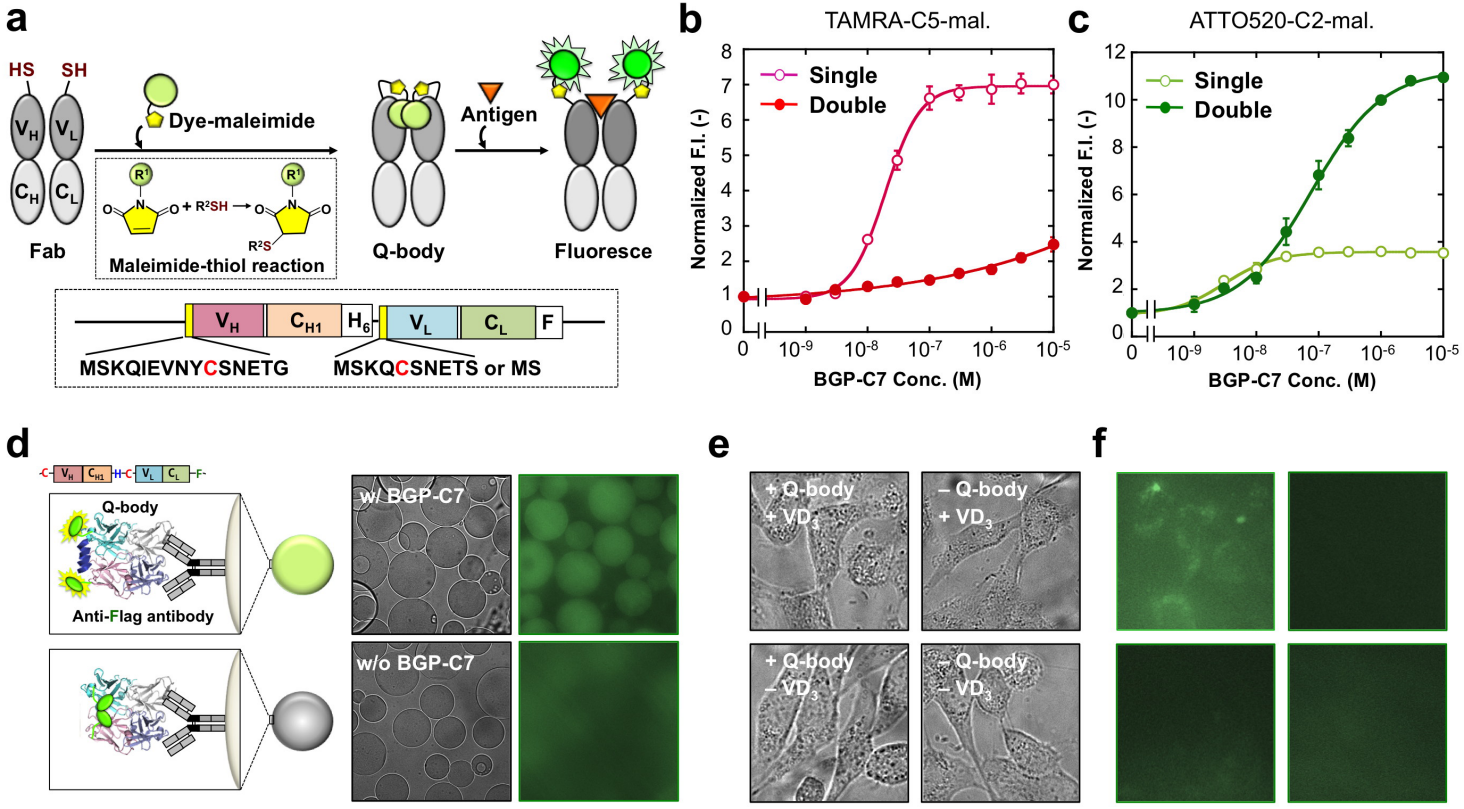
Preparation of UQ-bodies from *E. coli* cytoplasm. (a) Scheme of constructing UQ-bodies. Cys-tagged anti-BGP Fab fragment was expressed in *E. coli* SHuffle cells, and purified via the His_6_ tag at the C-terminus of the H (Fd) chain. After mild reduction of the exposed SH group, dye-maleimide was reacted and purified via the Flag tag at the C-terminus of the L chain. (b–c) Dose-response relationship of TAMRA-labeled (b) and ATTO520-labeled (c) UQ-bodies. An average with 1 SD of the three samples is shown. (d) Microscopic observation of anti-Flag M2 beads with and without double ATTO520-labeled UQ-body. The transmission and fluorescence images are shown. (e) Transmission and (f) fluorescence images of U2OS cells at the indicated conditions.

Although the UQ-bodies prepared using the *in vitro* translation system performed well, its relatively high cost and low yield prevents further applications, which require larger quantities of probe, such as cellular imaging (which needs sub-milligram amounts of probe). To resolve this issue, we prepared UQ-bodies using a combination of *E. coli* expression and thiol-based fluorescence labeling techniques (post-labeling method). To express the anti-BGP Fab fragment with one or two Cys-containing ProX tag(s) at the N-terminal region, bicistronic expression vectors encoding the tagged Fd and L chains were produced. To obtain soluble disulfide-bonded Ig domains in the *E. coli* cytoplasm, *E. coli* Shuffle (DE3) cells with an oxidized cytoplasm were transformed with the vector, and the cytoplasmic fraction was recovered after culture. After IMAC, mild reduction and fluorescence labeling were performed, followed by anti-Flag affinity purification, and sufficient amounts of UQ-bodies with high purity were obtained (Fig. 5a). In addition to TAMRA-C5-Maleimide, a shorter wavelength derivative ATTO520-C2-Maleimide was used for labeling, which exhibited less propensity for H-dimer formation. First, the antigen-binding activity of the double-labeled UQ-bodies was confirmed using ELISA with immobilized antigen (Supplementary Fig. S5a). We found that both TAMRA and ATTO520 double-labeled UQ-bodies showed high antigen-binding activity with similar Fab dose-dependency. In addition, each chain of the UQ-bodies showed sufficient purity and fluorescence, as assessed using SDS-PAGE (Supplementary Fig. S5b). It is worth noting that in the cases of single-labeled UQ-body, only a fluorescent band for heavy chain (Fd chain) was observed, ruling out the possibility of off-target labeling of intradomain Cys residues. When the fluorescence was evaluated using a fluorescence spectrometer, a significant antigen-dependent fluorescence increase was observed for single TAMRA- and double ATTO520-labeled UQ-bodies (Fig. 5, b and c). Specifically, double ATTO520-labeled UQ-body showed an 11-fold increase, and retained a low EC_50_ of 7.7 × 10^−8^ M. Also, single TAMRA-labeled UQ body showed a 7.0-fold increase with a lower EC_50_ of 1.9 × 10^−8^ M. In contrast, single ATTO520- and double TAMRA-labeled UQ-bodies showed a relatively modest antigen-dependent response of up to 3.6- and 2.4-fold, respectively. The contrasting responses of these two dyes probably reflect the differences in their quenching and dimerization behaviors (see discussion).

### Live imaging of BGP-producing osteoblasts using a UQ-body

Because UQ-bodies with a sufficient fluorescence response were obtained using the post-labeling method, microscopic antigen detection was performed. To achieve this, the most responsive double ATTO520-labeled UQ-body was applied to anti-Flag M2 beads, and its fluorescence was observed using a fluorescence microscope (Fig. 5d). We found that upon the addition of BGP-C7 peptide, the beads began to fluoresce, which was readily observed with visual inspection. Next, detection of the antigen on agarose beads was performed. When streptavidin-agarose beads were immobilized with or without biotinylated antigen (bioBGP-C11) peptide, and added with or without UQ-bodies, only the beads with both the antigen peptide and UQ-bodies showed intense fluorescence (Supplementary Fig. S6b).

Bone homeostasis is dependent on the balance of deposition by osteoblast and resorption by osteoclasts. This dynamic process is responsible for the continuous remodeling of bone tissue and is crucial for maintenance of bone size, shape, and integrity^12^. To identify osteoblasts in the tissue, markers such as bone-type alkaline phosphatase (BAP) are often used. However, conventional enzymatic assay for BAP was not specific enough, while typical immunoassay needs multiple steps for the detection. Since BGP represents a more specific mature osteoblast marker, imaging of BGPs produced by differentiated osteosarcoma cells was performed. Human osteosarcoma U2O2 cells were induced for differentiation to osteoblasts using 100 nM vitamin D_3_ (VD_3_). After incubation for 36 h, UQ-bodies were added to the cells, and directly observed using microscopy without washing. As shown in Fig. 5, e and f, a clear UQ-body-dependent fluorescence of VD_3_-treated cells was observed inside and/or around individual cells. Since most BGP secreted by normal osteoblast is deposited in extracellular bone matrix^13^, the observed intracellular fluorescence might represent internalized BGP due to added UQ-body. On the contrary, the U2OS cells not treated with VD_3_ showed lower fluorescence, probably reflecting lower production of BGP as reported^14^. Collectively, the result demonstrated the utility of the UQ-body as a handy live cell imaging/assay tool.

## Discussion

Our results suggested that the Fab demonstrated an enhanced response compared to the corresponding scFv in single TAMRA-labeled Q-bodies. This result reflected the more stable variable region structure of the Fab fragment. However, it was also equally possible that if the stability was too high, TAMRA could not penetrate into the V_H_/V_L_ interface where the Trp-mediated quenching occurs. Because the primary structure of our recombinant Fab is very close to its native counterpart, this result suggested that in natural antibodies, the transient opening and closing of the V_H_/V_L_ interface also occurs, which allows the entry of hydrophobic small molecules.

Compared with single labeled UQ-bodies, double-labeled UQ-bodies showed generally higher responses; however, TAMRA-labeled BGP UQ-bodies show a reduced sensitivity. In contrast, double ATTO520-labeled UQ-body demonstrated a higher sensitivity and response (Fig. 5b). This difference most likely reflected the dyes' similar yet distinct chemical structures (Supplementary Fig. S7). Compared with TAMRA, ATTO520 is less hydrophobic due to its smaller size and positive charge, and this resulted in its lower H dimer propensity. Conversely, double TAMRA-labeled UQ-bodies with different linker lengths (C0, C2 and C5) showed absorption peaks at a shorter wavelength, which indicated the presence of an H dimer (Supplementary Fig. S8). This result strongly supported a higher but difficult-to-release quenching of these UQ-bodies in the absence of antigen.

As a cellular imaging probe, we showed its utility in the extracellular imaging of live cells. However, if we inject the UQ-bodies into the cells by an appropriate method, clearer visualization of various intracellular targets and their modifications than previously reported with conventional fluorescence-labeled antibodies^15^ will be possible. Although optimization of the dye and its number might improve its performance, the observed potential to convert many antibody clones to UQ-bodies will innovate and redefine future immunodiagnostics, biosensing, and bioimaging studies.

## Methods

### Materials

The KOD-FX DNA polymerase was obtained from Toyobo, Osaka, Japan. The In-Fusion Advantage PCR Cloning Kit was obtained from Takara Bio (Otsu, Japan). Restriction enzymes and *E. coli* SHuffle T7 Express LysY were purchased from New England Biolabs (Ipswich, MA). The pIVEX2.3d vector was obtained from 5-Prime GmbH (Hamburg, Germany). The RYTS kit was purchased from ProteinExpress (Chiba, Japan). C-terminal peptides from BGP (BGP-C7, NH_2_-RRFYGPV-COOH, MW = 894) and biotinylated C-terminal peptides from BGP (BGP-C11, bio-NH_2_-QEAYRRFYGPV-COOH) were obtained from Genscript (Piscaway, NJ).

Ni Sepharose High Performance His beads were purchased from GE Healthcare (Piscataway, NJ). The Nanosep Centrifugal-10 k Ultrafiltration Device was obtained from Pall (Port Washington, NY). Immobilized TCEP Disulfide Reducing Gel and Streptavidin beads were purchased from Thermo Pierce (Rockford, IL). 5(6)-Carboxytetramethylrhodamine-C5-maleimide (TAMRA-C5-maleimide) and ATTO520-C2-maleimide were obtained from Biotium (Hayward, CA). TAMRA-C2-maleimide and TAMRA-C0-maleimide were obtained from Anaspec (Fremont, CA) and Life Technologies (Carlsbad, CA), respectively. Immunoblock was purchased from DS Pharma (Osaka, Japan). Anti-His-HRP antibody was purchased from Qiagen (Hilden, Germany). 3,3′,5,5′-Tetramethylbenzidine (TMBZ), anti-Flag M2 affinity gel, bovine and human serum albumins were obtained from Sigma (St. Louis, MO). A 35-mm glass bottom dish was obtained from Matsunami (Osaka, Japan). Dulbecco's Modified Eagle Medium (DMEM) and Vitamin D_3_ were purchased from Wako (Osaka, Japan). Fetal bovine serum (FBS) and Penicillin/streptomycin were obtained from Life Technologies. All water used was purified using Milli-Q (Millipore, UK). Other chemicals and reagents, unless otherwise indicated, were obtained from Sigma or Wako. The mouse IgG_1_ constant region gene was obtained from the HSRRB gene bank (Osaka, Japan). The mouse Ck gene derived of myeloma MOPC 41 was a kind gift from Klaus Rajewsky.

### Vector construction

An expression vector pROX-BGP-VH-CH1 harboring a T7 promoter-controlled heavy-chain Fd (VH-CH1) gene of anti-BGP C-terminal fragment KTM-219^7^ fused with an N-terminal ProX tag containing an amber codon (ATG TCT AAA CAA ATC GAA GTA AAC TAG TCT AAT GAG) and a C-terminal FLAG tag, was generated by splice-overlap-extension (SOE) PCR. The VH chain was amplified using the 5′-primer (CTTTAAGAAGGAGATATACCATGTCTAAACAAATCGAAGTAAACTAGTCTAATGAGACCCAAGTAAAGCTGCAGCAGTC) and 3′-primer (GATGGGGGTGTCGTTTTGGCGCTCGAGACGGTGAC), and the mouse IgG1 CH1 gene was amplified using the 5′-primer (GCCAAAACGACACCCCCATC) and 3′-primer (CTTTGTTAGCAGCCGGATCCTTATTACTTGTCATCGTCGTCCTTGTAGTCAGAACCCCCCCCACAATCCCTGGGCAC). The amplified V_H_ and C_H_1 genes were linked by SOEPCR using the V_H_ 5′-primer and CH1 3′-primer, and the construct was cloned into the *Nco*I- and *Bam*HI-digested pIVEX2.3d using the In-Fusion PCR cloning kit.

In addition, an expression vector pROX-BGP-VL-Cκ harboring a T7 promoter-controlled light-chain (VL-Cκ) gene of the KTM-219 fused with an N-terminal ProX tag containing an TTT codon and C-terminal His tag, was constructed by SOE PCR. The VL chain was amplified using the 5′-primer (CTTTAAGAAGGAGATATACCATGTCTAAACAAATCGAAGTAAACTTTTCTAATGAGACCGACATTGAGCTCACCC) and 3′-primer (GTTGGTGCAGCATCAGCCCGTTTTATTTCC), and the mouse Cκ gene was amplified using the 5′-primer (GCTGATGCTGCACCAAC) and 3′-primer (TGATGATGAGAACCCCCCCCACACTCATTCCTGTTG). The amplified V_L_ and Cκ genes were linked by SOE PCR using the V_L_ 5′-primer and the Cκ 3′-primer, and the construct was then cloned into *Nco*I- and *Sma*I-digested pIVEX2.3d using the In-Fusion PCR cloning kit.

The wild-type (non-labeled) Fab gene was constructed by replacing the TAG codon with a TTT codon in the ProX tag of VH-CH1. For doubly and FRET pair-labeled Fabs, the corresponding expression vectors were constructed by replacing the TAG with a TAG and CGGG codon, respectively, in the ProX tag preceding the VL-Cκ gene.

For Fab genes encoding anti-SA(29IJ6)^16^, morphine^17^, methamphetamine^18^, cocaine^19^ and influenza virus hemagglutinin (HA)^11^, the corresponding genes were cloned in place of the anti-BGP Fab, with an additional sequence encoding N-terminal (G_3_S)_2_ linker. The codon-optimized V_H_/V_L_ genes for anti-methamphetamine, cocaine and influenza HA were synthesized by Genscript (Piscaway, NJ, USA).

### Preparation of aminoacyl tRNAs

5-R110-C6-AF-pdCpA, 5(6-)TAMRA-C6-AF-pdCpA and ATTO655-C6-AF-pdCpA were synthesized as previously described^20^. The aminoacylated pdCpAs were ligated to an amber suppressor tRNA derived from *Mycoplasma capricolum* Trp1 tRNA or a four-base CGGG suppressor tRNA derived from yeast phenylalanine tRNA without the 3′ dinucleotide using chemical ligation method as previously described^21^,^22^. The aminoacyl-tRNAs can be obtained as commercially available reagents (CoverDirect tRNA reagents for site-directed protein labeling, ProteinExpress, Chiba, Japan).

### Cell-free co-transcription/translation

The incorporation of TAMRA-C6-AF into the N-terminal region of Fab was performed using a cell-free transcription/translation system. The reaction mixture (60 μL) consisted of 30 μL of the Reaction Mix (×2), 0.6 μL methionine, 3 μL Enzyme Mix, 20 μL *E. coli* lysate, each 2 μL plasmid DNAs (200 ng) and 3 μL aminoacyl-tRNA (480 pmol). All the reagents used, with the exception of the plasmid and tRNA, were provided in the RYTS kit. The reaction mixture was incubated at 37°C for 2 h, and subsequently at 4°C for 16 h. To obtain the FRET-pairs of labeled Q-body (HRLT), for example, a FRET-pair containing two fluorescent amino acids, TAMRA-tRNA_CUA_ and R110-tRNA_CGGG_ were added to the cell-free translation system combined with two expression vectors with a TAG codon in the ProX tag of the VH-CH1 gene and CGGG codon in the ProX tag of VL-Cκ gene.

An aliquot of the reaction mixture (0.5 μL) was applied to 15% SDS-PAGE and the gel was visualized using a fluorescence scanner FMBIO-III (Hitachi, Tokyo, Japan). The gel was also evaluated using Western blot analysis with anti-Flag M2 and alkaline phosphatase-labeled anti-mouse IgG (Promega, Madison, WI, USA) or anti-His tag (Novagen, La Jolla, CA, USA) and alkaline phosphatase-labeled anti-mouse IgG (Promega, Madison, WI, USA).

### Tandem affinity purification with anti-Flag- and nickel-affinity chromatography

First, the reaction mixture (60 μL) was incubated with 20 μL of Flag M2 affinity gel. After incubation at room temperature for 15 min, the column was washed three times with wash buffer (20 mM phosphate, 0.5 M NaCl, 0.1% polyoxyethylene(23)lauryl ether, pH 7.4). The bound proteins were subsequently eluted with two 200-μL volumes of wash buffer containing 100 μg/mL of Flag peptide. The eluted proteins were then applied to a His Spin Trap Column (GE Healthcare, Piscataway, NJ, USA). After incubation at room temperature for 15 min, the column was washed three times with wash buffer containing 60 mM imidazole. The bound proteins were then eluted with two 200-μL volumes of wash buffer containing 0.5 M imidazole. The eluate was subsequently passed through an UltraFree-0.5 centrifugal device (Millipore, Billerica, MA) and equilibrated with phosphate buffered saline supplemented with Tween-20 (PBST, 10 mM phosphate, 137 mM NaCl, 2.7 mM KCl, 0.05% Tween-20, pH 7.4) to concentrate the protein in the buffer. The concentration of the labeled Fab protein was determined by comparing the fluorescence intensities of a known concentration of free TAMRA- (Anaspec) and the sample under denaturing conditions in 7 M GdnHCl, 100 mM DTT, pH 7.4.

### Fluorescence measurements

For fluorescence spectral measurements of anti-BGP Fab, the purified Fab (70 nM, 6.25 μL) was mixed with various concentrations of the antigen in PBST (50 μL) containing 1% BSA. After incubation at 25°C for 70 min, the fluorescence spectra were measured in a 2 × 2 × 15 mm quartz cell (Starna, Atascadero, CA).

Anti-bovine SA, anti-morphine, anti-methamphetamine, anti-cocaine or anti-influenza virus HA Fab was mixed with various concentrations of antigen in PBST (50 μL) containing 1% BSA. The fluorescence spectra were immediately measured in a cuvette.

Fluorescence spectra for single or double TAMRA-labeled Q-bodies were measured from 565 to 700 nm with excitation at 550 nm, for R110-labed Q-bodies, from 515 to 650 nm with excitation at 480 nm, and for ATTO655-labed Q-bodies, from 655 to 800 nm with excitation at 640 nm at 25°C on a FluoroMax-4 (Horiba Jobin-Yvon, Kyoto, Japan). Fluorescence spectra for UQ-bodies hetero-labeled with R110 and TAMRA, or with TAMRA and ATTO655, were measured from 515 to 700 nm or from 565 to 800 nm, with excitation at 480 nm or 530 nm, respectively. The excitation and emission slit widths were set to 15.0 nm. The EC_50_ values were calculated using curve fitting of the observed fluorescence intensities at the maximum emission wavelength, and employing a sigmoidal dose-response model based on ImageJ software (http://rsbweb.nih.gov/ij/). All the fluorescence intensities were normalized taking the intensity of each sample at zero dose (without antigen) as one, at the maximum emission wavelength unless otherwise stated.

### Fluorescence-based thermal shift assay

The thermal shift assay was performed using the StepOne real-time PCR system (Applied Biosystems). For the thermal shift assay of the Q-body, the purified Fab was added to a final concentration of 70 nM in a 30 μL PBST containing 1% BSA. The change in fluorescence was monitored using the ROX filter. The temperature was increased from 25 to 98°C in 1°C intervals over the course of 1 min, with fluorescence readings taken at each interval. The fluorescence data were plotted, normalized, and the first derivative of the curve was calculated to provide the melting temperature *T*_m_.

### *E. coli* Expression and Purification of Fab fragment

Details for constructing the single and double Cys-tagged Fab expression plasmids pUQ1H(KTM219) and pUQ2(KTM219) will be described elsewhere. SHuffle T7 Express LysY cells were transformed with either pUQ1H(KTM219) or pUQ2(KTM219), and the transformed cells were cultured at 30°C for 16 h in LB medium containing antibiotics (100 μg/mL ampicillin) (LBA) and 1.5% agar. A single colony was picked and grown at 30°C in 4 mL of LBA medium until the OD_600_ reached 0.9, from which 1.6 mL was used to inoculate 100 mL of LBA medium. The cells were cultured at 30°C until the OD_600_ reached 0.6, when 0.4 mM isopropylthio-β-galactopyranoside was added and the solution was further incubated for an additional 16 h at 16°C. The solution was centrifuged (6,000 × *g*, 15 min, 4°C) and the pellet was resuspended in 5 mL HS buffer (20 mM phosphate, 0.5 M sodium chloride (NaCl), 60 mM imidazole, 0.1% polyoxyethylene(23)lauryl ether, pH 7.4) and then sonicated. After centrifugation (11 k*g*, 20 min, 4°C), the supernatant was subsequently purified as described below.

### Preparation of the UQ-body from recombinant Fab fragment

The Poly-Prep column (Bio-Rad) packed with 0.4 mL of Ni Sepharose High Performance His beads was primed with 2 mL of HS buffer. The synthesized protein was then applied to the column and washed five times with 2 mL of HS buffer. Finally, the proteins were eluted twice with 2 mL of elution buffer (20 mM phosphate, 0.5 M NaCl, 0.5 M imidazole, 0.1% polyoxyethylene(23)lauryl ether, pH 7.4). The eluent was subjected to a Nanosep Centrifugal-10 k Ultrafiltration Device and equilibrated with TCEP buffer (50 mM sodium phosphate, 100 mM NaCl, 10 mM EDTA, pH 7) to exchange buffers. The expression and purification of the protein were confirmed using SDS-PAGE analysis. The protein concentration was determined using the Bradford assay with BSA as a standard.

A volume of immobilized TCEP Disulfide Reducing Gel slurry (Thermo) equal to the volume of purified protein was added to a microtube and centrifuged at 50 × *g* for 1 min. After removing the supernatant, 1 mg of purified protein was added and incubated for 1 h at room temperature on a rotating wheel. After centrifuging for 1 min, the supernatant was recovered and its concentration was determined using the Bradford assay (Bio-Rad) with BSA as a standard.

The reduced protein was labeled with TAMRA-maleimide or ATTO520-maleimide in the dark for 2 h at 25°C. The molar ratio of protein to dye was one to two. The product was subsequently subjected to a Nanosep Centrifugal-10 k Ultrafiltration Device and equilibrated with PBST (0.05% Tween 20 in phosphate buffered saline, 10 mM phosphate, 137 mM NaCl, 2.7 mM potassium chloride, 0.05% Tween 20, pH 7.4) to exchange buffers and to remove unbound dye, which was confirmed using SDS-PAGE.

### Antigen binding ELISA

After 100 μL of streptavidin (10 μg/mL in PBS) was immobilized on a Falcon 3912 microplate (BD Bioscience, Tokyo, Japan) for 2 h at 25°C, the well was filled with PBS containing 20% Immunoblock for 2 h at 25°C, and washed three times with PBST. Subsequently, 100 μL of biotinylated BGP-C11 (4 ng/mL in PBST containing 20% Immunoblock) was added and incubated for 1 h at 25°C. After washing three times with PBST, each of the concentrations of fluorolabeled protein, “UQ-body”, in 100 μL of PBST containing 5% Immunoblock was added and incubated for 2 h at 25°C. The well was washed three times with PBST and bound UQ-body was probed with 100 μL of 2,000-fold diluted Anti-His-HRP antibody in PBST containing 5% Immunoblock for 1 h at 25°C. The well was washed three times with PBST, and developed with 100 μL of substrate solution (100 μg/mL TMBZ and 0.04 μL/mL hydrogen peroxide, in 100 mM sodium acetate, pH 6.0). After incubation for 5 min, the reaction was stopped with 50 μL of 1 M sulfuric acid, and the absorbance was determined at 450 nm with a reference at 655 nm using a microplate reader Model 680 (Bio-Rad, Tokyo, Japan).

### Fluorescence measurement

To measure the fluorescence spectra, 100 ng of UQ-body was diluted in 250 μL of PBST containing 1% BSA, and antigen BGP-C7 peptide was added by titration in a 5 × 5 mm quarts cell (GL Sciences, Tokyo, Japan). After each addition, the solution was incubated for 5 min at 25°C prior to the spectral measurements. As a control, the same procedure was performed with the exception that PBST was added instead of BGP-C7 peptide in the cell. The fluorescence spectra were obtained at 25°C using a fluorescence spectrophotometer Model FP-8500 (JASCO, Tokyo, Japan) with the excitation at 546 and 524 nm for TAMRA- and ATTO520- UQ-body, respectively. The excitation and emission slit widths were set to 5.0 nm. The dose-response curves were fitted at the maximum emission wavelength using Kaleida Graph 4.1 (Synergy Software, Reading, PA).

### Imaging of agarose beads with UQ-body

To image the immobilized UQ-body on the beads, 10 μL of Anti-Flag M2 affinity gel and 100 ng of double ATTO520-labeled UQ-body were incubated for 30 min at room temperature on a rotating wheel. After washing with PBS to remove unbound UQ-body, 0–1 μg of BGP-C7 was added. The sample was subsequently placed on a 35-mm glass bottom dish, and imaged using a fluorescence microscope IX71 (Olympus, Tokyo, Japan) equipped with 470 ± 20 nm excitation and 520 ± 20 nm emission filters. The images were obtained using the HCImage system equipped with an ImagEM EM-CCD camera (Hamamatsu Photonics, Japan).

To image the antigen on the beads, streptavidin agarose beads (10 μL) and 1 μg of biotinylated BGP-C11 peptide were incubated for 30 min at 25°C on a rotating wheel. After washing by centrifugation (1,000 × *g*, 1 min, 4°C) to remove unbound peptide, 100 ng of double ATTO520 labeled UQ-body was added, followed by washing with PBST. After the addition of 250 μL PBST, the sample was placed on a 3.5-cm dish, and imaged. Controls without biotinylated BGP-C11 peptide or UQ-body were employed.

### Imaging of osteocalcin produced by human osteoblasts

U2OS cells were seeded onto 35-mm glass bottom dishes and cultured to 70% confluency in DMEM supplemented with 10% FBS and 1% P/S at 37°C with 5% CO_2_. The cells were washed three times with PBS and incubated in serum-free DMEM for 24 h and then incubated for 36 h in DMEM containing 0.1% FBS and 10^−7^ M vitamin D_3_. Double ATTO520-labeled UQ-body (100 ng) was added and was imaged. The exposure time was 1 s. As a control, the same procedures without the addition of vitamin D_3_ and/or UQ-body were used.

## Author Contributions

R.A. and H.U. conceived of the study; R.A., H.T. and H.U. designed the experiments; H.J.J., D.A., K.Y. and J.D. performed the experiments in *E. coli*; R.A., R.K. and H.O. performed the experiments *in vitro*; F.S. performed the narcotics measurement; R.A. and H.J.J. analyzed the results; and R.A. and H.U. wrote the manuscript with input from H.T.

## Supplementary Material

Supplementary Information Supplementary Information

## Figures and Tables

**Table 1:** Antigen-dependent fluorescence enhancements and EC_50_ of double-labeled Fab type BGP Q-bodies

	Dye		Detection		
	H chain	L chain	*E*_x_/*E*_m_	Normalized FL intensity at λ_max_	EC_50_
HTLT	TAMRA	TAMRA	530/580	21.2*	1.0 × 10^−4^ M
HTLn	TAMRA	-	530/580	9.6	2.1 × 10^−8^ M
HnLT	-	TAMRA	530/580	1.8	7.1 × 10^−9^ M
HRLR	R110	R110	480/530	2.4	7.6 × 10^−9^ M
HRLn	R110	-	480/530	1.1	7.8 × 10^−9^ M
HnLR	-	R110	480/530	1.3	7.2 × 10^−9^ M
HALA	ATTO655	ATTO655	630/680	10.6	4.4 × 10^−8^ M
HALn	ATTO655	-	630/680	4.6	4.6 × 10^−9^ M
HnLA	-	ATTO655	630/680	2.7	9.8 × 10^−9^ M

*Fluorescence intensity not saturated.

**Table 2:** Antigen-dependent fluorescence enhancements and EC_50_ of FRET pairwise-labeled Fab-type BGP Q-bodies

	Dye		Detection		
	H chain	L chain	*E*_x_/*E*_m_	Normalized FL intensity at λ_max_	EC_50_
HTLR	TAMRA	R110	480/530	2.7	8.8 × 10^−9^ M
			480/580	2.5	9.7 × 10^−9^ M
HRLT	R110	TAMRA	480/530	8.0	1.2 × 10^−7^ M
			480/580	19	1.1 × 10^−7^ M
HTLA	TAMRA	ATTO655	530/580	35	6.1 × 10^−8^ M
			530/680	9.4	1.3 × 10^−7^ M
HALT	ATTO655	TAMRA	530/580	50	1.1 × 10^−7^ M
			530/680	2.5	9.7 × 10^−9^ M
