# Supplementary material for: Ultra Q-bodies: quench-based antibody probes that utilize dye-dye interactions with enhanced antigen-dependent fluorescence
Source: Sci Rep. 2014 Apr 11;4:4640. doi: 10.1038/srep04640 (PMC3983608; doi:10.1038/srep04640)

Supplementary information

Ultra Q-bodies : quench-based antibody probes that utilize dye-dye interaction show enhanced antigen-dependent fluorescence

Authors: Ryoji Abe[1], Hee-Jin Jeong[2,3], Dai Arakawa[2], Jinhua Dong[2,3], Hiroyuki Ohashi[1,2], Rena Kaigome[1], Fujio Saiki[1], Kyosuke Yamane[1,2], Hiroaki Takagi[1], and Hiroshi Ueda[2,3,*]

[1]Ushio Inc., 6409 Moto-Ishikawa-cho, Aoba-ku, Yokohama, Kanagawa 225-0004, Japan

[2] Department of Chemistry and Biotechnology, School of Engineering, The University of Tokyo, 7-3-1 Hongo, Bunkyo-ku, Tokyo 113-8656, Japan.

[3]Chemical Resources Laboratory, Tokyo Institute of Technology, 4259-R1-18, Nagatsuta-cho, Midori-ku, Yokoyama, Kanagawa 226-8503, Japan.

* Correspondence should be addressed to H.U. (ueda@res.titech.ac.jp)

Supplementary Table S1

	Dye		Detection	Normalized FL intensity at λ_{max}	EC_{50} (mg/mL)
	H chain	L chain	E_x / E_m		
H5N1/HTLT	TAMRA	TAMRA	530 / 580	3.3	6.5
H5N1/HTLR	TAMRA	R110	480 / 530	6.1	2.6
H5N1/HRLT	R110	TAMRA	480 / 530	2.8	1.7
H5N1/HTLA	TAMRA	ATTO655	530 / 580	2.6	4.3
H5N1/HALT	ATTO655	TAMRA	530 / 580	4.2	1.2
H1N1/HTLR	TAMRA	R110	480 / 530	7.1	0.7

Supplementary Figure Legend

Figure S1. Thermal shift assay. (a) Scheme of the temperature-dependent removal of a quenching effect by denaturation of Quenchbody. (b) Temperature-dependent fluorescence intensity. The temperature was raised from 25 to 98°C at 1°C intervals per minute, with fluorescence readings taken at each interval. (c) Melt curve obtained as a differential of (b), showing the peaks corresponding to the apparent melting temperature T_m.

Figure S2. Antigen-dependent fluorescence enhancement of anti-BGP Fab single/double-labeled with R110 (a and b) or ATTO655 (c and d). (a) Standard curves of the fluorescence intensity at 530 nm. The Fabs labeled with R110 at indicated position(s) were excited at 480 nm in the absence and presence of BGP-C7 peptide. The intensities are relative values with respect to that in the absence of BGP-C7 peptide. (b) Normalized standard curves. (c) Standard curves of the fluorescence intensity at 680 nm. The Fab labeled with ATTO655 was excited at 630 nm. (d) Normalized standard curves.

Figure S3. Antigen-dependent fluorescence enhancement of R110/TAMRA-heterolabeled Fabs. (a) Fluorescence spectra of anti-BGP Fab labeled at the N-terminal region of the H chain with R110 and the L chain with TAMRA (HRLT), with excitation at 530 nm in the presence of BGP-C7 peptide as indicated. (b) The same with Fab labeled with TAMRA at the H chain and with R110 at the L chain (HTLR). (c) Standard curves of the fluorescence intensity at 580 nm. The intensities are relative values with respect to that in the absence of BGP-C7 peptide. (d-f) The results of anti-SA UQ-bodies for detecting human serum albumin. The conditions are the same as in (a-c), except that HSA was used as an antigen and the fluorescence intensity at 530 nm was taken in (f).

Figure S4. Detection of Influenza virus HA proteins by hetero-labeled UQ-bodies. (a) Standard curves of the fluorescence intensity at 530 nm. The intensities are relative values with respect to that in the absence of H5N1 HA (A/Vietnam/1194/04). Abbreviations for the labeled Fabs are the same as in other figures. (b) Fluorescence spectra of anti-HA UQ-body labeled with TAMRA at Fd and with R110 at L chain (HTLR), with excitation at 485 nm in the presence of H5N1 HA protein as indicated. (c) Comparison of the standard curves for H5N1 and H1N1 (A/California/04/09) with HTLR-type UQ-body. (d) Fluorescence spectra as shown in (b), except that H1N1 protein was used.

Figure S5. Characterization of UQ-bodies. (a) Antigen-binding activity of the double-labeled UQ-bodies. Biotinylated BGP-C11 (1 μg/ml) was immobilized through streptavidin, and varied amounts of labeled Fab fragments were added, which was probed by HRP-conjugated anti-His Ab. (b and c) SDS-PAGE showing the purified UQ-bodies. Single (s) or double (d) labeled UQ-bodies are visualized by their fluorescence (b) or after CBB-staining (c).

Figure S6. Detection of bead-bound antigen by double ATTO520-labeled UQ-body under a Fluorescence microscopy. Detection scheme (upper), pictures taken by transmission (middle) and fluorescence (lower) are shown.

Figure S7. Working model of double-labeled UQ-bodies. Less hydrophobic ATTO520 (upper left) has a net positive charge and is easily exposed to outside of the Fab after antigen binding. More hydrophobic TAMRA has higher tendency to form H dimer, which is difficult to dissolve upon antigen binding.

Figure S8. Effect of linker length between TAMRA and maleimide. (a) Dose-response of double TAMRA UQ-bodies with three different linker lengths (0, 2, and 5). (b) Normalized absorption spectra of free maleimide dyes (b) and of the UQ-bodies at 510 nm. The ratio of two absorption peaks at shorter (~520 nm) and longer (~550 nm) shows relative amount of H-dimer.

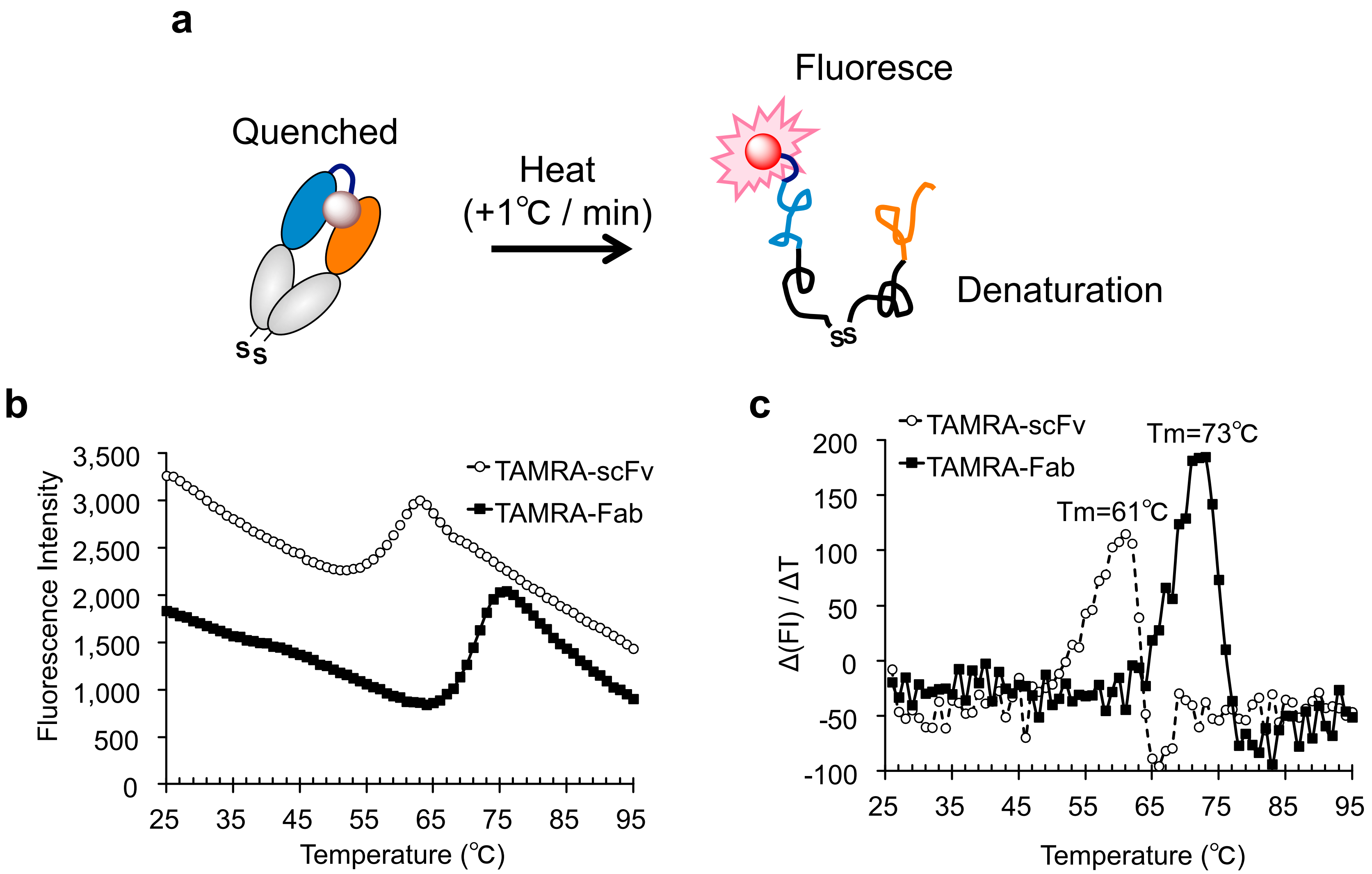
a
Quenched
Heat
(+1℃ / min)
Fluoresce
Denaturation
S S
S S
b
Fluorescence Intensity
3,500
3,000
2,500
2,000
1,500
1,000
500
0
25 35 45 55 65 75 85 95
Temperature (℃)
TAMRA-scFv
TAMRA-Fab
c
Δ(FI) / ΔT
200
150
100
50
0
-50
-100
25 35 45 55 65 75 85 95
Temperature (℃)
TAMRA-scFv
TAMRA-Fab
Tm=61℃
Tm=73℃

Supplementary Figure S2

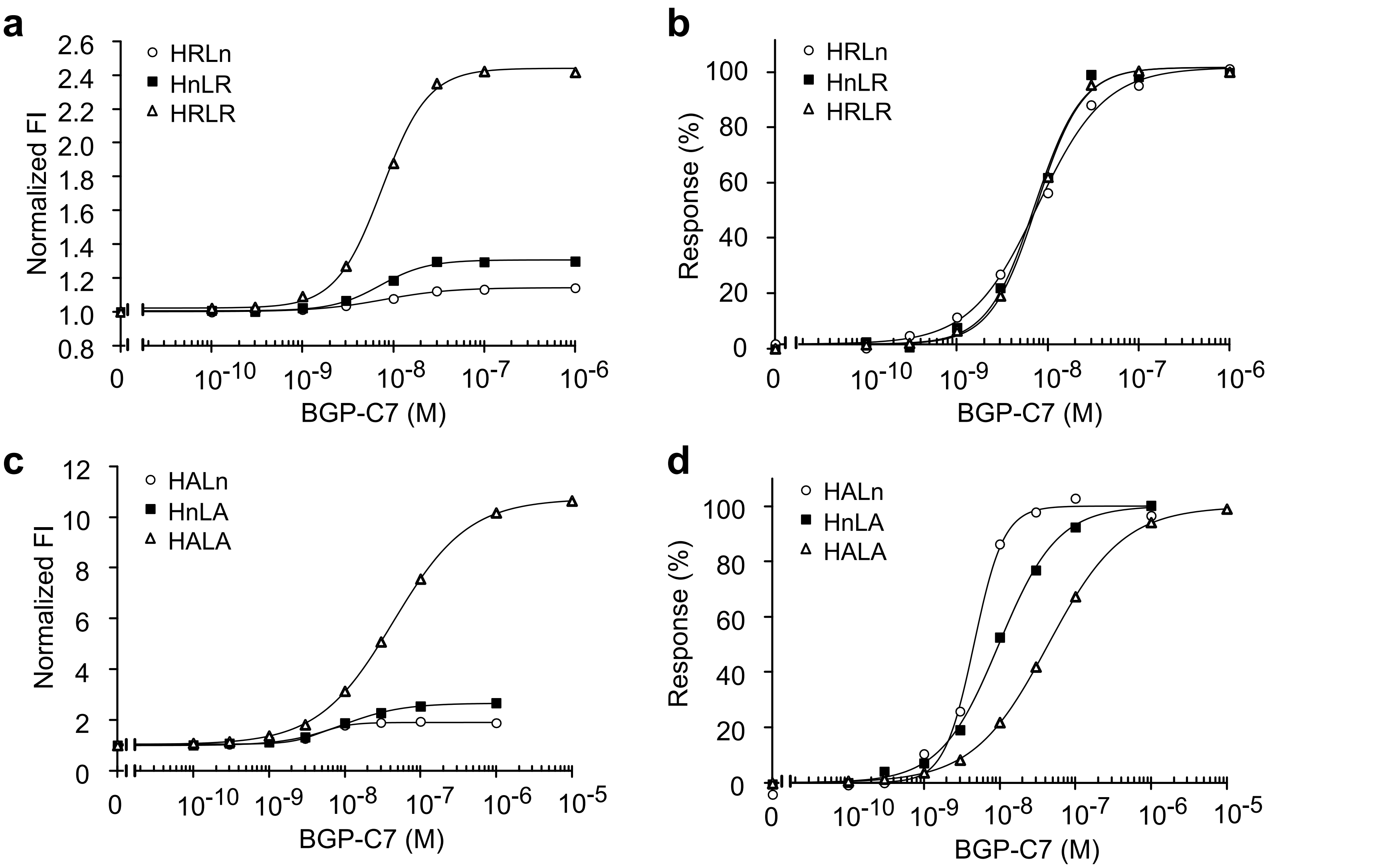

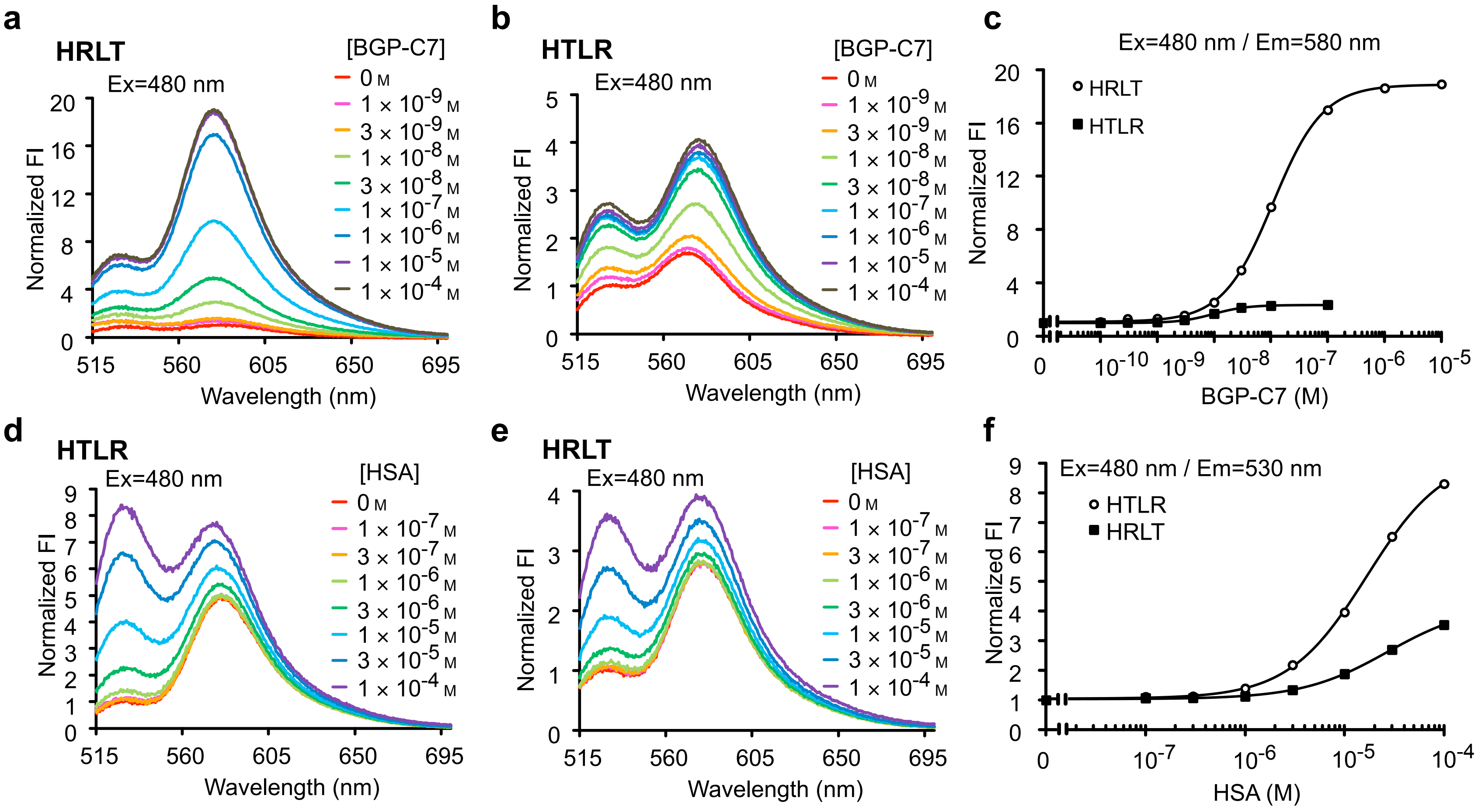
a
HRLT
Ex=480 nm
[BGP-C7]
0 M
1 × 10-9 M
3 × 10-9 M
1 × 10-8 M
3 × 10-8 M
1 × 10-7 M
1 × 10-6 M
1 × 10-5 M
1 × 10-4 M
Normalized FI
Wavelength (nm)
b
HTLR
Ex=480 nm
[BGP-C7]
Normalized FI
Wavelength (nm)
c
Ex=480 nm / Em=580 nm
HRLT
HTLR
Normalized FI
BGP-C7 (M)
d
HTLR
Ex=480 nm
[HSA]
0 M
1 × 10-7 M
3 × 10-7 M
1 × 10-6 M
3 × 10-6 M
1 × 10-5 M
3 × 10-5 M
1 × 10-4 M
Normalized FI
Wavelength (nm)
e
HRLT
Ex=480 nm
[HSA]
Normalized FI
Wavelength (nm)
f
Ex=480 nm / Em=530 nm
HTLR
HRLT
Normalized FI
HSA (M)

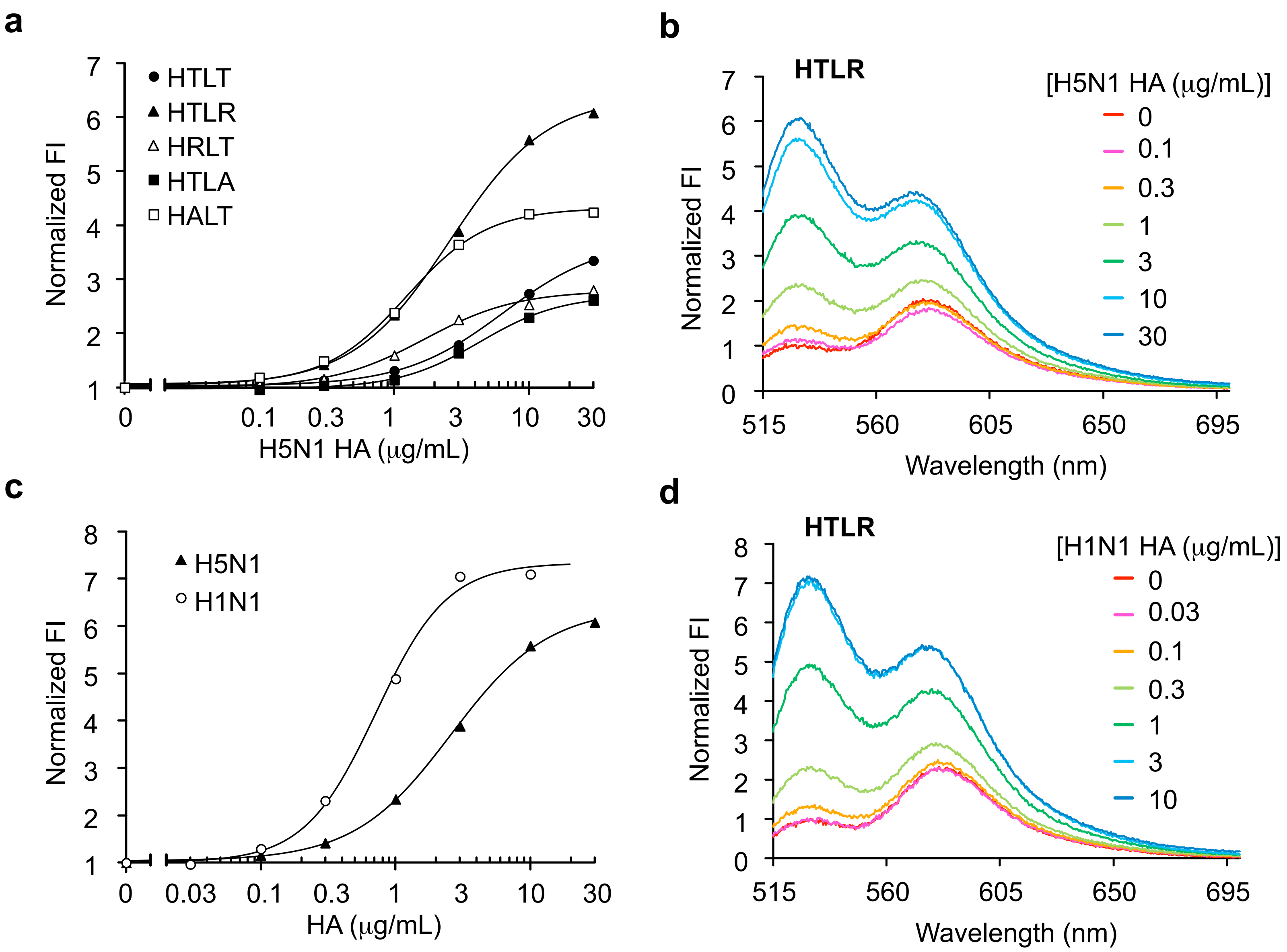
a
Normalized FI
HTLT
HTLR
HRLT
HTLA
HALT
H5N1 HA (μg/mL)
b
HTLR
[H5N1 HA (μg/mL)]
0
0.1
0.3
1
3
10
30
Normalized FI
Wavelength (nm)
c
H5N1
H1N1
Normalized FI
HA (μg/mL)
d
HTLR
[H1N1 HA (μg/mL)]
0
0.03
0.1
0.3
1
3
10
Normalized FI
Wavelength (nm)

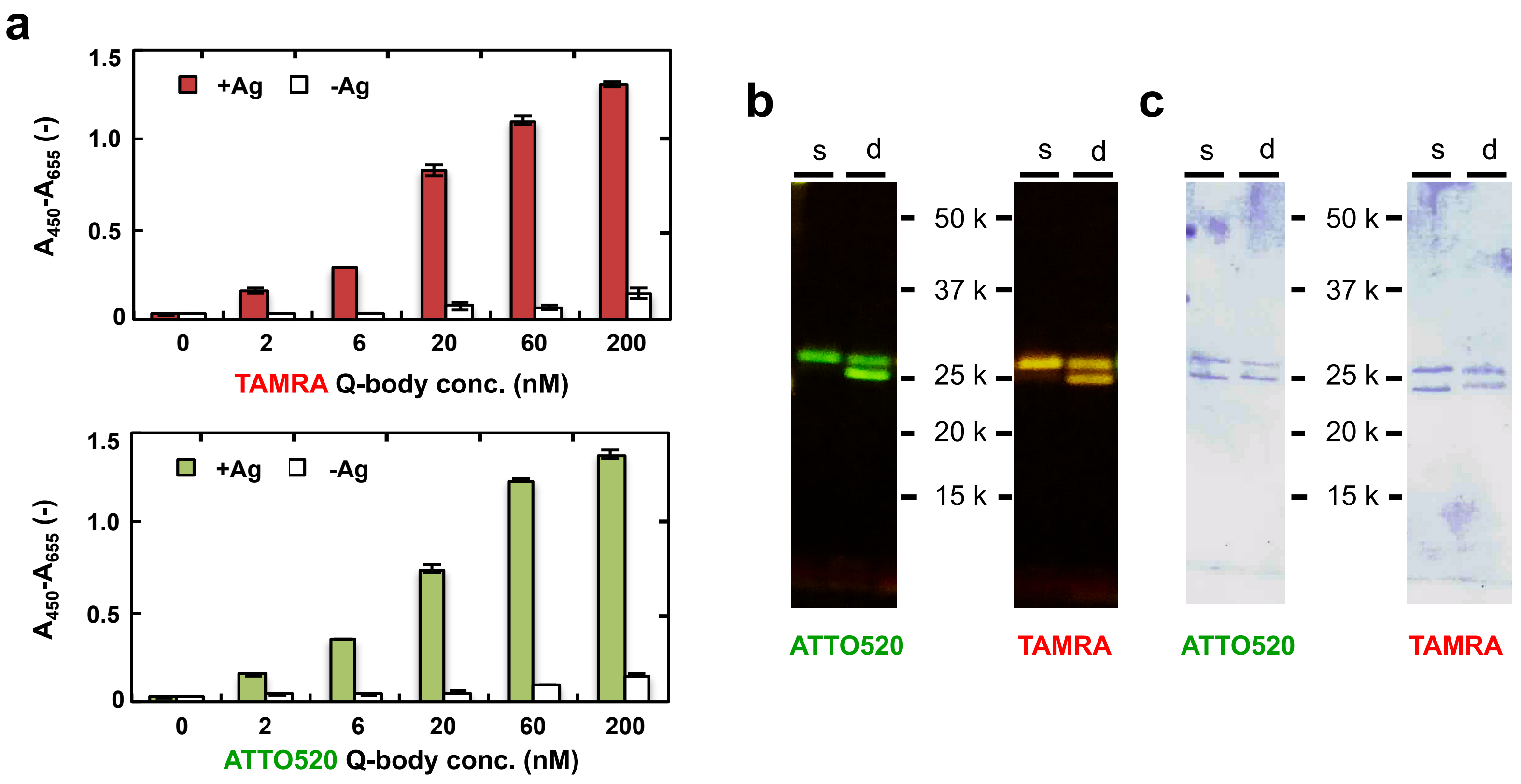
a
1.5
1.0
0.5
0
+Ag
-Ag
A450-A655 (-)
0
2
6
20
60
200
TAMRA Q-body conc. (nM)
ATTO520 Q-body conc. (nM)
b
c
s
d
50 k
37 k
25 k
20 k
15 k
ATTO520
TAMRA

Supplementary Figure S6

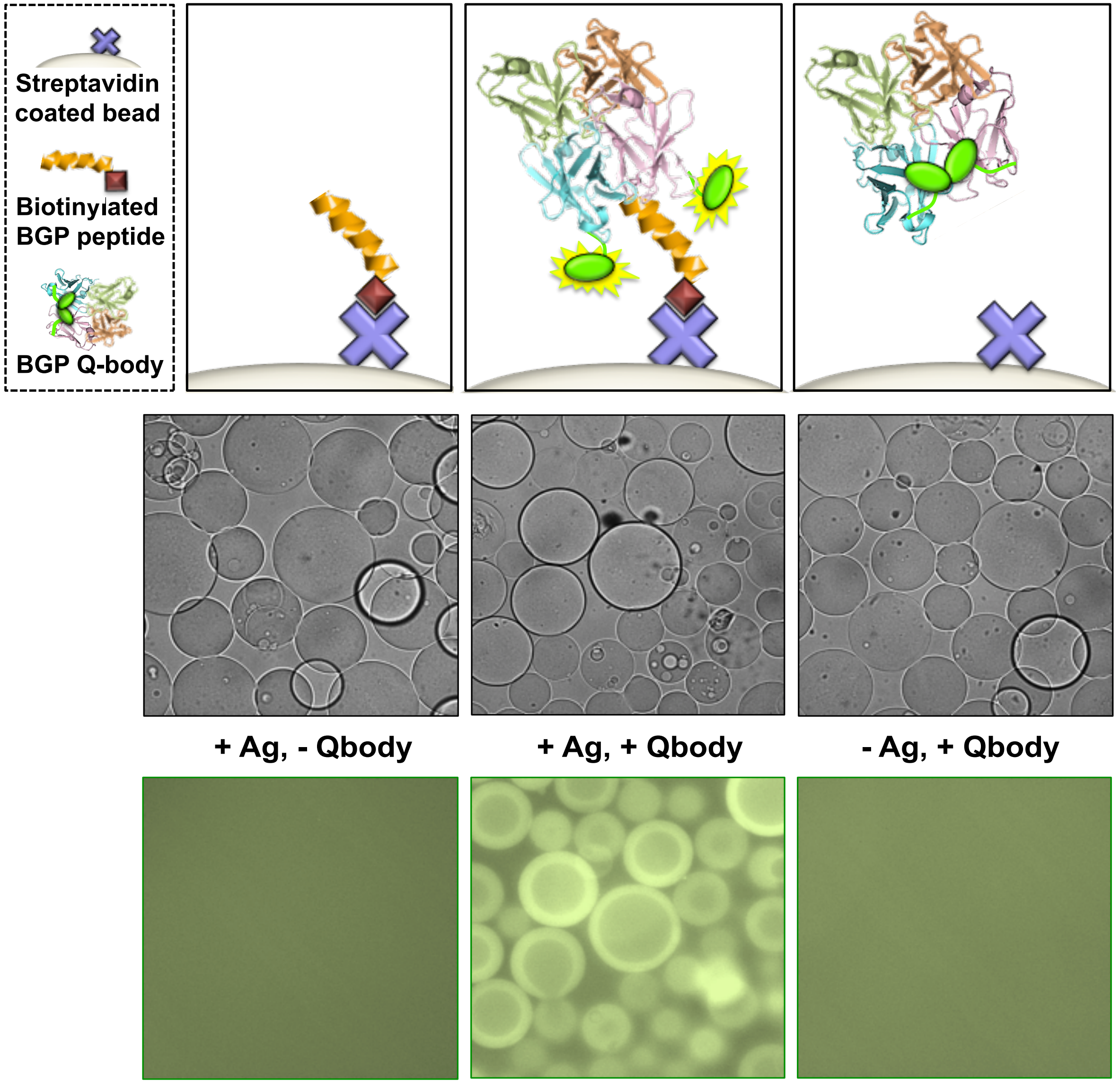

Supplementary Figure S7

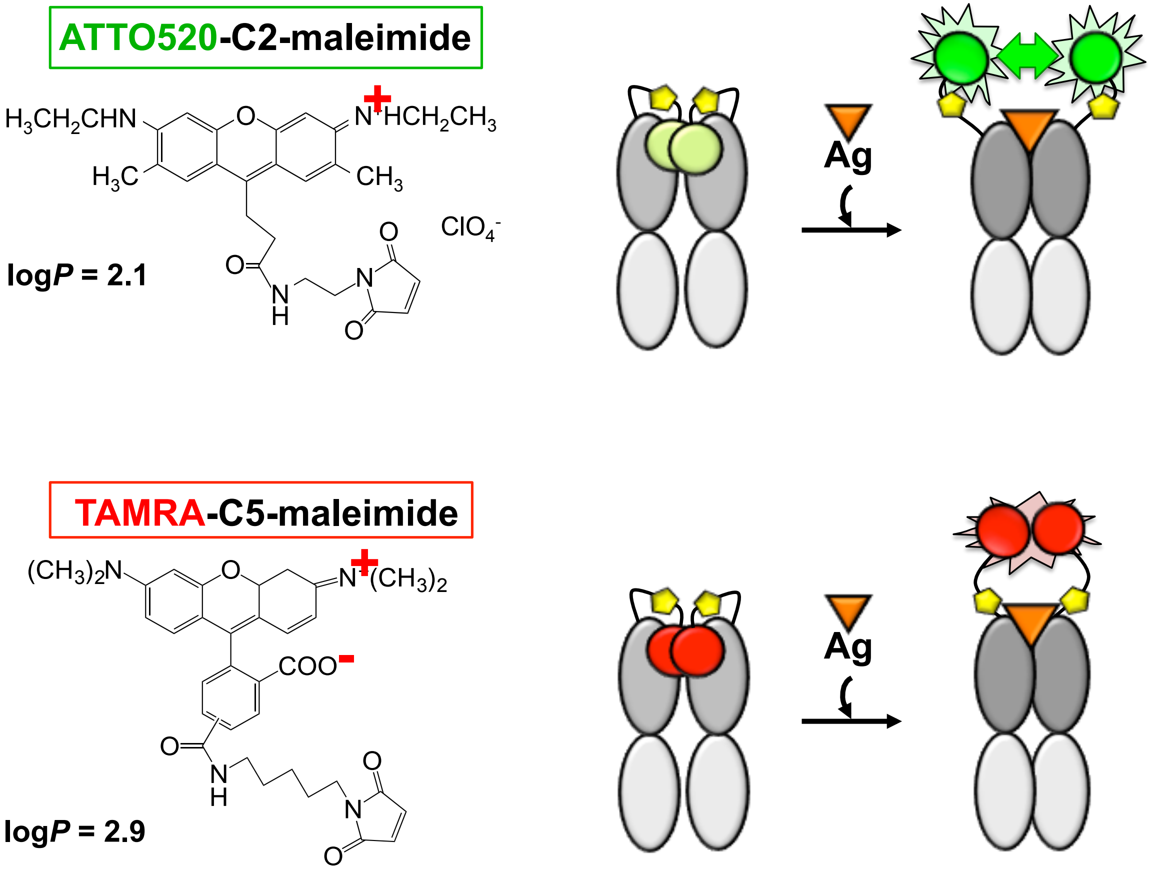

Supplementary Figure S8

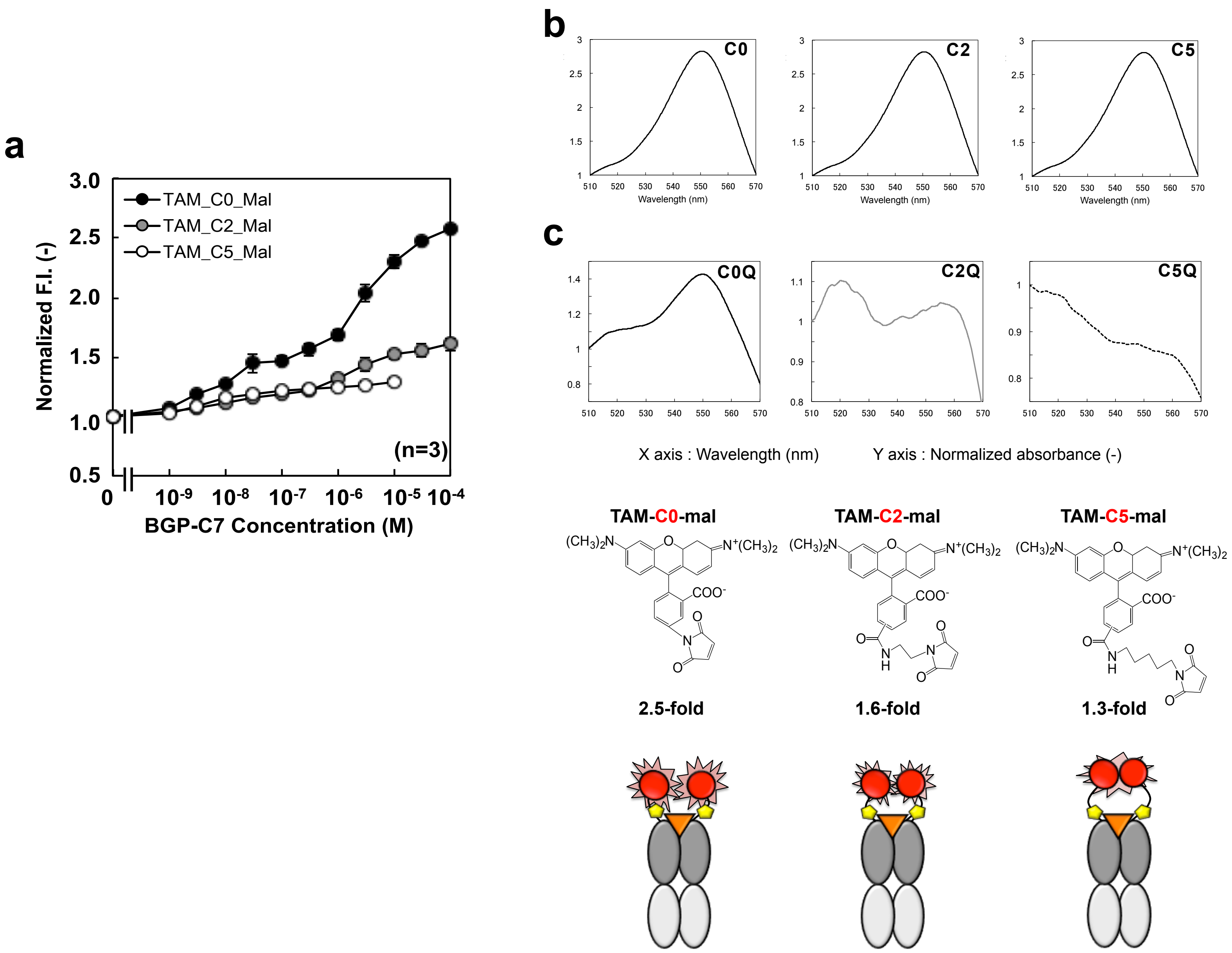

Supplement: Supplementary Information [file srep04640-s1.pdf]
